# High heterogeneity of fecal carriage extended-spectrum beta-lactamase-producing *E. coli* isolated from iranian community and clinical settings

**DOI:** 10.1186/s12879-022-07304-7

**Published:** 2022-03-31

**Authors:** Shadi Aghamohammad, Vajihe Sadat Nikbin, Farzad Badmasti, Fereshteh Shahcheraghi

**Affiliations:** Department of Bacteriology, Pasteur Institute of Iran, Tehran, Iran

**Keywords:** Fecal carriage, *Escherichia coli*, Clonal relatedness

## Abstract

**Background:**

Extended-spectrum beta-lactamase-producing enterobacteria (ESBL-PE) in carriers have become a global health problem. Using molecular typing techniques, including PFGE, could be useful to determine the source of bacterial dissemination. The current study aimed to investigate the intestinal carriage of ESBL-producing *E. coli* (ESBL-EC) and clonal relatedness among ESBL-EC isolated from hospitalized and outpatient fecal carriers in Iran.

**Methods:**

A total of 120 rectal swabs were collected; 50.8% (61/120) from intensive care unit (ICU) inpatients and 49.2% (59/120) from outpatients. MacConkey agar enriched with cefotaxime was used to screen the ESBL-EC. PCR assays were performed to detect ESBL and carbapenemase genes. Pulse-fields gel electrophoresis (PFGE) was performed to assess clonal relatedness.

**Results:**

Totally, 60.0% (72/120) were carrier for ESBL-EC. The rates of resistance against ceftazidime and cefepime were 90.2% (65/72) and 93.0% (67/72), respectively. The rates of *bla*_CTX-M-15_, *bla*_TEM_, *bla*_SHV_, *bla*_NDM-1_, *bla*_OXA-48_ and *bla*_IMP_ was 90.2% (65/72), 50.0% (36/72), 5.5% (4/72), 4.1% (3/72), 4.1% (3/72) and 1.3% (1/72), respectively. Based on a cut-off 80%, 69 ESBL-EC isolates could be categorized in 10 mini-cluster and 47 isolates were considered as singletons.

**Discussion:**

High heterogeneity among isolates from ESBL-EC suggests that this bacterium probably has a different source of dissemination. Screening of carriers in hospitals and communities could help the infection control program in public health.

**Supplementary information:**

The online version contains supplementary material available at 10.1186/s12879-022-07304-7.

## Background

Distribution of Extended-Spectrum beta-lactamase-producing *Enterobacteriaceae* (EPE), first described in 1983. The distribution of EPE become widespread, these days. The location of the ESBL coding gene in transposable elements, including plasmids, leads to easier transmission of these genes [1]. Besides clinical samples, EPE has recently become more notable in the fecal carriage. Antibiotic consumption and traveling abroad specifically to Africa and Asia are mentioned as important risk factors to become a fecal carriage [2]. Fecal carriages often show the slightest clinical symptoms, however, the presence of carriers in hospital settings could be notable in the aspect of transmission [3]. Fecal carriages in hospitalized patients could be a threatening challenge, since the transmission of resistance genes among patients could exacerbate the situation, however, detecting EPE carriers in a healthy population has the same importance. If the rate of fecal carriage in the community and healthy person be controlled, the rate of carriers in hospital settings also could be decreased [4].

The presence of ESBL, including CTX-M and TEM among *Enterobacteriaceae* could lead to serious infections both in the hospital and community. The rate of ESBL coding genes in *E.coli* was reported high in different studies and was considered as a major public health problem [5]. Investigation of the rate of ESBL-producing *E. coli* (ESBL-EC) among hospitalized and outpatient carriers is critical to control the spread of resistant strains in both community and hospital settings. The presence of these genes in silent carriers could increase the rate of resistant infections without alarming prognostic symptoms [6]. Furthermore, most of the other genes related to aminoglycoside and fluoroquinolone resistance are located in the same plasmids; therefore, the transmission of these plasmids leads to cause multi-drug resistance [7]. The ESBL prevalence rate in Iran and other eastern countries such as Pakistan and China is high, and recently this rate has increased significantly due to self-medication and overuse of third-generation cephalosporins in hospitals [8, 9]. Among different members of *Enterobacteriaceae*, *E. coli* is one of the most commonly producing ESBL bacteria, and its specific phylogenic ones, such as phylogroup B2 and ST131 are associated with global dissemination [10]. Phylogroup B2 and F are considered as the sources of clinical infections [11]. Therefore, finding these phylogroups in fecal carriages of EPE could be notable and considered as an alarmingly high-risk factor to cause further clinical infections in carriers.

Besides investigation about the rate of fecal carriages of EPE, using molecular typing techniques, including PFGE could be useful to determine the source of dissemination of these isolates [12]. Using PFGE as a gold standard typing method to understand the clonal relatedness among isolates and comparing the other data, including the phylogroup types and antibiotic distribution, could help the health care system to follow the sources of infection and possible resistance genes distribution.

Up to now, there were few reports about the rates of fecal carriages of EPE in Iran [3, 7, 13, 14]. Most of the studies were focused more on clinical samples. Investigation about the fecal carriers in more detail and showing the clonal relatedness of the isolates could be impressive in infection control. The current study aimed to investigate the intestinal carriage of ESBL-EC and clonal relatedness among ESBL-EC isolated from hospitalized and outpatient fecal carriers.

## Material and method

### Ethical statement and patient sampling

The present cross-sectional study was conducted from January to October 2016 in a general university hospital in Tehran, Iran. A total of 120 rectal swabs (RS) were randomly collected from ICU and outpatients. All methods were carried out in accordance with relevant guidelines and regulations and ethical approval is obtained from the committee of Pasteur Institute of Iran (IR.PII. REC.1395.44). Sign informed consent was obtained from all participants. The datasets generated and analyzed during the current study are not publicly available due to their proprietary nature, privacy, and ethical concerns, but are available from the corresponding author on reasonable request.

### Phenotypic identification of fecal carriage

To deliver rectal swabs to the laboratory within 2 h, Tryptic Soy Broth containing a 30-µg cefotaxime (CTX) disc (Mast Group Ltd., Merseyside, UK) was used as a transport media [3]. All swabs were incubated overnight at 37 °C. To recognize the fecal carriages in the first step, MacConkey agar supplemented with cefotaxime (1 mg/L) was used [7]. Every growing strain was considered as a resistant one and to get a pure culture, a single colony was sub-cultured on the Mac-Conkey agar supplemented with cefotaxime and then the *E. coli* strains were confirmed by biochemical tests [15].

### Antimicrobial susceptibility test

The antibiotic susceptibility of cefotaxime-resistant *E. coli* isolates was determined by using the Kirby-Bauer disk diffusion susceptibility test according to the Clinical and Laboratory Standards Institute (CLSI) guidelines (CLSI, 2017). The susceptibility against eleven antibiotics, including ceftazidime (CAZ: 30 µg), ceftazidime/clavulanic acid (CAZ/CLA: 30/10 µg), cefotaxime (CTX: 30 µg), cefotaxime/clavulanic acid (CTX/CLA: 30/10 µg), cefepime (CPM: 30 µg), amikacin (AK: 30 µg), gentamicin (GM: 30 µg), ciprofloxacin (CIP: 5 µg), levofloxacin (LVX: 5 µg), ertapenem (ETP: 10 µg), imipenem (IMP: 10 µg) (all from MastGroup Ltd., Merseyside, United Kingdom) were examined. *E. coli* ATCC 25922 was used as a control sample. The double-disk synergy test (DDST) was performed according to the CLSI guidelines to detect ESBL producing isolates. *Klebsiella pneumoniae* ATCC 700603 and *E. coli* ATCC 25922 were used as positive and negative controls in the DDST method, respectively. Every strain that was resistant against at least one agent in three or more antibiotic classes, considered as the Multi-Drug Resistant (MDR).

### Molecular detection of ESBL and carbapenemase genes

Genomic DNA was extracted according to a DNA extraction kit (Bioneer Company, Korea, AccuPrep Genomic DNA Extraction Kit). Polymerase chain reaction (PCR) was performed to identify ESBL and carbapenemase genes, including *bla*_TEM_, *bla*_SHV_, *bla*_CTX-M-15_, *bla*_VEB_, *bla*_PER_, *bla*_NDM-1_, *bla*_OXA-48_, *bla*_VIM_, and *bla*_IMP_. Primer sequences were described, previously [16].

### Pulse field gel electrophoresis (PFGE)

The ESBL-EC strains were subjected to PFGE to analyze the clonal relatedness Genomic DNA of ESBL-EC isolates and the reference marker *Salmonella enterica* serotype Braenderup strain H9812 was digested with the endonuclease XbaI and then the genomic bands were separated using a CHEF-DRIII system (Bio-Rad Laboratories) as described, previously [17]. A similarity ratio was determined using Dice coefficients. Cluster analysis was performed using the unweighted pair group method with arithmetic means (UPGMA). DNA fragment analysis was performed using Gelcompar II (V.4.1) (Applied Maths, Belgium). Isolates that had a similarity cut-off ≥ 80% of their banding patterns were considered to belong to the same clonal lineage (cluster).

### Statistical analysis

Statistical analysis of the data was performed using SPSS (version 25; SPSS, Inc., Chicago, IL, USA). Statistical differences were evaluated via the Chi-square test and the odds ratio. *P*-values* <* 0.05 were considered statistically significant.

## Results

### Bacterial isolates

From 120 non-duplicated rectal swabs, no bacterial isolate was detected among 20 swabs. The rate of ESBL-EC from a total of 120 rectal swabs, was 60.0% (72/120). 50.8% (61/120) of isolates were gained from inpatients of intensive care unit (ICU) and 49.2% (59/120) were collected from outpatients. According to the present results, 55.5% (40/72) of outpatients and 45.4% (32/72) of inpatients were carriers of ESBL-EC. Out of 72 ESBL-EC carriers, 39 (54.1%) were men and 33 (45.8%) were women. Demographic data of ESBL-EC carriers and non-carriers were summarized in Table 1. The additional data of each patient could be seen in Additional file 1: Tables S1 and S2. According to the odds ratio test, there was no significant difference in the history of antibiotic usage, hospitalization, surgery, traveling abroad, and underlying disease between ESBL-EC carriers and non-carriers (*p* > 0.05).

**Table 1:** Demographic data of carriers and non-carriers of ESBL-producing *E. coli*

Carrier/non-carriers of ESBL	Risk factors								
	Gender		Antibiotic usage within the past 6 months	Hospitalization within the past 6 months	Underlying disease	Antibiotic usage within the past 6 months in the family members	Hospitalization within the past 6 months in the family members	Surgery	Traveling abroad
	Male	Female							
Carriers of ESBL-producing *E. coli*	39/72 (54.1%)	33/72 (45.8%)	30/72 (41.6%)	10/72 (13.8%)	22/72 (30.5%)	13/72 (18%)	6/72 (8.3%)	9/72 (12.5%)	6/72 (8.3%)
Non-carriers of ESBL-producing *E. coli*	8/20 (40%)	12/20 (60%)	9/20 (45%)	2/20 (10%)	5/20 (25%)	1/20 (5%)	1/20 (5%)	3/20 (15%)	1/20 (5%)

### Antimicrobial susceptibility

The results of the Kirby-Bauer disk diffusion test showed that the highest resistance rates of ESBL-EC isolates were against ceftazidime and cefepime [90.2% (65/72) and 93.0% (67/72), respectively]. The rates of resistance to ciprofloxacin, levofloxacin, gentamicin, amikacin, ertapenem, and imipenem were 44.4% (32/72), 36.1% (26/72), 15.2% (11/72), 1.3% (1/72), 4.1% (3/72), 2.7% (2/72), respectively. About 15.2% (11/ 72) of ESBL-EC isolates were MDR. Fortunately, resistance to carbapenem was restricted to strains isolated from ICU patients. The rate of MDR was significantly higher in patients from ICU in comparison to outpatients (*p* < 0.05).

### Carbapenemase and ESBL genes

Totally, according to the present data, the rates of the *bla*_CTX-M-15_, *bla*_TEM_, and *bla*_SHV_ were 90.2% (65/72), 50.0% (36/72), and 5.5% (4/72), respectively. The *bla*_NDM-1_, *bla*_OXA-48_ and *bla*_IMP_ genes were detected in 4.1% (3/72), 4.1% (3/72) and 1.3% (1/72) of isolates. No isolates were positive for the *bla*_KPC_, *bla*_VIM_, *bla*_VEB_, and *bla*_PER_ genes. The epidemiological data of the cases with carbapenemase genes were listed in Table 2. ESBL and carbapenemase coding genes were detected both in outpatients and patients from ICU. All ESBL-EC isolates harbored at least one ESBL gene and 6.9% (5) of isolates had both ESBL and carbapenemase genes. There was no statistical difference between the history of antibiotic usage within the past 6 months in the fecal carriers or their family members and the presence of more than one resistance gene among carriers in this study (*p*-value > 0.05).

**Table 2:** Epidemiological data of cases with carbapenemase genes

Code	Epidemiological data									
	Gender	Age	Units	Antibiotic usage within the past 6 months	Hospitalization within the past 6 months	Underlying disease	Antibiotic usage within the past 6 months in the family members	Hospitalization within the past 6 months in the family members	Surgery	Traveling abroad
E69	F	65	G-ICU	+	−	−	−	−	−	−
E65	M	61	G-ICU	−	−	+	−	−	−	−
E71	F	64	E-ICU	+	−	+	−	−	+	−
E28	M	39	OP	−	−	−	−	−	−	−

*F* female, *M *male, *ICU* intensive care unit, *OP* outpatient, *G-ICU* general intensive care unit, *E-ICU* emergency intensive care unit

### Clonal relationship of ESBL-EC isolates

**Fig. 1:**
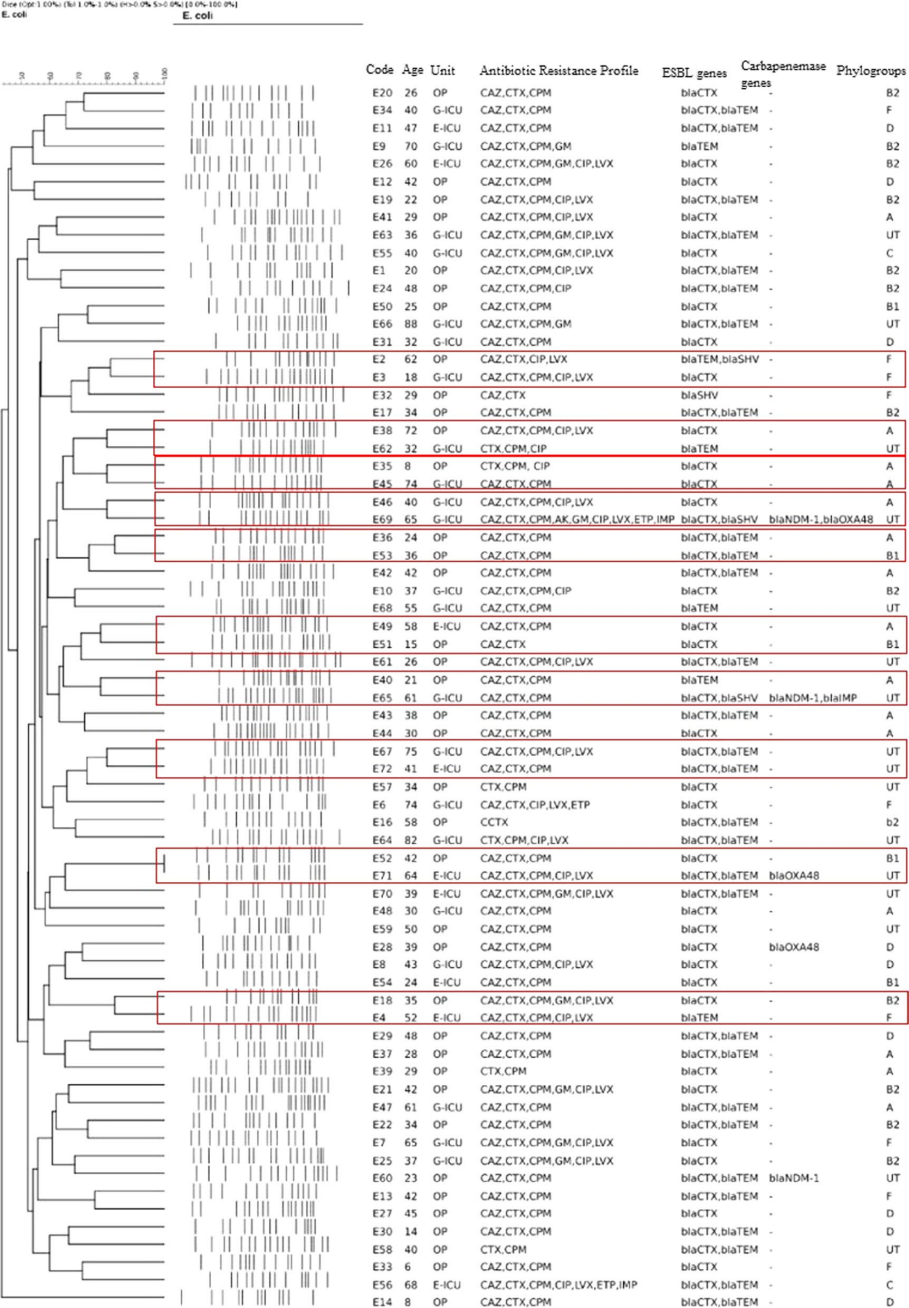
Clonal relatedness, antimicrobial resistance pattern, phylogroups, and gene distribution among ESBL-EC fecal carriages. **The mini-clusters are determined with red rectangles. The other strains are singletons**. *ICU* intensive care unit, *OP* Outpatient, *G-ICU* general intensive care unit, *E-ICU* emergency intensive care unit, *CAZ* ceftazidime, *CTX* cefotaxime, *CPM* cefepime, *AK* amikacin, *GM* gentamicin, *CIP* ciprofloxacin, *LVX* levofloxacin, *ETP* ertapenem, *IMP* imipenem

Based on a cut-off of 80% genetic similarity, PFGE revealed high heterogeneity among ESBL-EC isolates. According to the present study, three isolates were reported to be untypeable (after repeating three times) due to the degradation of DNA during the procedure. 69 ESBL-EC isolates could be categorized in 10 mini-clusters, while 49 isolates appeared to be singletons. The clonal relatedness, gene distribution, and antimicrobial resistance pattern among ESBL-EC carriers were summarized in Fig. 1.

## Discussion

Clinical infections caused by *Enterobacteriaceae* are investigated through the past years. The presence of the resistance genes among these isolates is challengeable since it could cause treatment failure. ESBL production is one of these resistance mechanisms. The cephalosporines, specifically third and fourth generations, are extended-spectrum antibiotics. The presence of resistance mechanisms in bacterial agents against these antimicrobial drugs could make the treatment more difficult [18].

Producing ESBL in *Enterobacteriaceae* is such a challenge that the Centers for Disease Control and Prevention (CDC) classifies *Enterobacteriaceae* as hazard level, the level in which vancomycin-resistant *Enterococcus* (VRE), Methicillin-resistant *Staphylococcus aureus* (MRSA), and MDR/XDR tuberculosis are also classified [19]. On the other hand, the resistance genes are located in the transposable elements, including plasmids; therefore, they have the propensity to spread easily between humans (hand carriage, contaminated food, and water, medical equipment) [20]. Along with clinical infections, the fecal carriages of EPE are now becoming important, since some studies report the high increase (even up to tenfolds) in the rate of carriers in the last years [21]. The use of antibiotics, history of hospitalization, traveling abroad specifically to the area with a high prevalence of clinical infections and fecal carriages, and animal contact are some of the risk factors associated with fecal carriages [22].

One of the important points is that colonization of EPE in the gastrointestinal tract rarely causes clinical symptoms; therefore, the carriage is hardly ever noted by the health care system. However, being a fecal carrier could lead to host infection, the transmission of resistance genes to other bacterial species, and also dissemination to other individuals specifically the family members [23]. Host infection (due to prior colonization) is an important point in fecal carriages. According to Goulenok et al., the median time from colonization to infection could be as short as 12.5 days, specifically in patients hospitalized in ICU [24] and the duration of being a fecal carriage could be lasted up to 1 year [25].

Besides determining the rate of fecal carriages, evaluating the characteristics of strains isolated from fecal carriages could be important. For instance, using typing methods to show the clonal relatedness between different strains, assessing antibiotic resistance profile, resistant genes distribution, are some critical factors that should be assessed in fecal carriages. In the present study, among 120 non-duplicated rectal swabs, the rate of ESBL-EC was 60.0% (72/120). According to Bezabih et al., the increased trend in fecal carriages from 2.6% in 2003–2005 to 21.1% in 2015–2018 has been detected [26]. Our result was similar to the rate of fecal carriages in India (72%) and Southeast Asia (60%) [27]. Besides, in the current study, the rate of cephalosporine resistance was as high as approximately 90% and 15.2% (11/ 72) of ESBL-EC isolates were MDR. The most prevalent ESBL gene was *bla*_CTX-M15_; furthermore, all ESBL-EC isolates harbored at least one ESBL gene and 6.9% (5) of isolates had both ESBL and carbapenemase genes. As can be seen, the rate of resistance was high among our fecal carriages. Another important note in the current study is that the rate of ESBL-EC among outpatients (55.5%) was a little higher than in ICU patients (45.4%). The presence of unknown fecal carriage in the nuclear family could be considered as one of the causes of easily transmitted resistant genes and increase the rate in the community. Moreover, considering other risk factors, including the high-risk occupations, including slaughterhouse workers, may affect the rate of ESBL-EC in the community. This remarkable data emphasizes the need to control the resistant gene distribution in the community. Although some risk factors such as antibiotic usage, history of hospitalization, surgery, and traveling abroad were evaluated in the present study, no significant result could obtain, except for the rate of MDR between outpatients and patients from ICU. The presence of MDR isolates and carbapenem resistance in fecal carriages in our ICU patients is significant data. One reason may be because the usage of carbapenem antibiotics is often restricted in hospitals according to the antibiotic stewardship program (ASP) that restricts carbapenems usage in ICU, and also other wards of the hospital, [28] and another reason could be due to the high rate of antibiotic usage (in different classes) in the hospital settings in our country. On the other hand, evaluation of risk factors among isolates that were in the same mini-clusters revealed that there was little similarity among these strains. Only E49 and E51 had the same history of antibiotic use, E40 and E65 had the underlying disease, and E18 and E4 both had a history of surgery. Also, the rate of positive risk factors, including surgery or hospitalization history did not differ between isolates that were in mini-cluster and those that were singletons. It should be noted that due to the high heterogeneity of our isolates, the low similarity of risk factors between isolates from the same mini-cluster or strains that were singletons was to be expected.

Another noteworthy piece of information that should be discussed is the fact that extended-spectrum cephalosporins, fluoroquinolones, and carbapenems are widely used for treatment or prophylaxis in our country, especially in the hospital setting [29]. The high consumption could lead to an increase in ESBL rates because, as mentioned earlier, antibiotic overusing is one of the most important risk factors to become a fecal carriage.

The presence of MDR isolates in ICU is critical since these strains could shift to clinical infections, and therefore make the situation of the hospitalized patient worse. Besides, using PFGE as a typing method, showed high heterogeneity among studied isolates. In a previous study conducted by our team, phylogroup typing, MLST, and plasmid replicon typing were performed [7]. Gathering the data of the past and the present results showed notable points. Although the PFGE results showed high heterogeneity (10 mini clusters and 47 singletons), evaluation of the other characteristics among isolates that were in the same mini-cluster revealed remarkable points. E2 and E3, for instance, belonged to two patients of different ages (a 62-year-old man as an outpatient and an 18-year-old man as a hospitalized patient in general-ICU). As can be seen in Fig. 1, these two isolates were in the same mini-cluster. Evaluation of the MLST and phylogroup typing results (performed in the past study) revealed that both these isolates had phylogroup F (one of the members of putative virulent phylogroups) with the same ST (ST769). The same status was also seen in strains E35 and E45. These strains were isolated from an 8-year-old outpatient boy and a 74-year-old hospitalized woman in general-ICU with a different history of antibiotic usage and hospitalization. Both of these strains belonged to phylogroup A (commensal phylogroup). These findings revealed some critical issues. First, both typing techniques in our research, including MLST and PFGE, highlighted the same clonality among these strains and their final results confirmed each other. Second, phylogroup typing revealed that both of the isolates in each cluster showed probably the same source. Third, having the same ST and PFGE pattern in two strains which are isolated from two completely separate individuals (one from the outpatient group and the other from hospitalized patients) showed the circulation of a single strain both in the community and hospital. On the other hand, we had some strains, E67 and E72 for instance, that were isolated from two patients hospitalized in general and emergency-ICU, or E46 and E69 that were isolated from two patients in general-ICU. This could show the probable circulation of a single strain in the hospital that could affect other patients and health care staff through dissemination. Another important note about strain E69 was the high rate of antimicrobial resistance and gene distribution in this strain. Since it seems that E46 and E69 had the same origin, the probability of getting a resistance profile in E46 also could be increased. Furthermore, detecting plasmid type of IncFI and IncFII (in E18 and E4 which were belonged to the same mini cluster) showed the high probability of ESBL genes dissemination, since several studies indicated that IncF incompatibility group (IncFII and IncFI) are associated with the dissemination of CTX-M-15 [30]. Totally, it could be said that the rate of fecal carriages, specifically in healthy individuals has been more investigated in Iran, recently. According to Hajihasani et al., the rate of ESBL-EC in healthy fecal carriages in 2018 was 43.1% (233/540). Similar to our results, the most prevalent ESBL gene in their study was *bla*_CTX-M15_ (93.9%) and also PFGE results showed high heterogenicity. One of the important notes in the mentioned study was the detection of ST131 and the presence of ceftazidime/avibactam (CAZ/AVI)-resistant *E. coli* in the healthy carriers [31]. The presence of ESBL/carbapenemase-producing ST131 in healthy carriers could exacerbate the status of clinical infection in the community.

In conclusion, our study provides novel information about the presence and distribution of the EPE isolates along with clonal relatedness in fecal carriages in Iran. Several reasons could be important in causing the high rate of EPE intestinal colonization among carriers: (i) lack of proper management of infection control and screening of carriers at an early stage of admission, (ii) lack of health monitoring in some high-risk occupations, including restaurant staff (both in hospital and community), (iii) close relationship between n Iranian people and neighboring countries, and (iv) uncontrolled prescription of antibiotics. The ESBL-EP fecal colonization rate is remarkable in some neighboring countries, including Afghanistan [32] and also the rate of immigrants from those regions, is high in our country. Therefore, using monitoring protocols to identify the fecal carriages specifically in border areas could be so important to decrease the rate of fecal carriage in our country. The presence of resistant strains in the silent reservoir could increase the risk of clinical infections both in community and clinical settings.

## Supplementary Information

**Additional file 1: Table S1.** Demographic data of ESBL-producing *E. coli *fecal carriages. **TableS2.** Demographic data of non-carriers of ESBL-producing *E.coli*.

## Data Availability

The datasets generated and analyzed during the current study are not publicly available due to their proprietary nature, privacy, and ethical concerns, but are available from the corresponding author on reasonable request.
