# Supplementary material for: High heterogeneity of fecal carriage extended-spectrum beta-lactamase-producing E. coli isolated from iranian community and clinical settings
Source: BMC Infect Dis. 2022 Mar 31;22:318. doi: 10.1186/s12879-022-07304-7 (PMC8973555; doi:10.1186/s12879-022-07304-7)
Supplement: Supplementary file 1 — Additional file 1: Table S1. Demographic data of ESBL-producing E. coli fecal carriages. TableS2. Demographic data of non-carriers of ESBL-producing E.coli. [file 12879_2022_7304_MOESM1_ESM.docx]

**Table S1. Demographic data of ESBL-producing *E. coli* fecal carriages.**

| Code | Gender | Antibiotic usage within the past 6 months | Hospitalization within the past 6 months | Underlying disease | Antibiotic usage within the past 6 months in the family members | Hospitalization within the past 6 months in the family members | Surgery | Traveling abroad |
| --- | --- | --- | --- | --- | --- | --- | --- | --- |
| E20 | M | - | - | - | - | - | - | - |
| E34 | F | - | - | - | - | - | - | - |
| E11 | M | + | - | - | - | - | - | - |
| E9 | M | + | + | + | - | - | - | - |
| E26 | M | + | + | - | - | - | - | - |
| E12 | M | + | - | - | - | - | - | - |
| E19 | F | + | - | + | + | - | - | - |
| E41 | F | + | - | - | + | - | - | - |
| E63 | M | + | + | - | - | - | + | - |
| E55 | F | - | - | + | - | - | - | - |
| E1 | M | + | + | - | + | - | + | - |
| E24 | F | + | - | + | + | - | - | + |
| E50 | M | - | - | - | - | - | - | - |
| E66 | F | + | + | + | - | - | - | - |
| E31 | M | - | - | - | - | - | - | - |
| E2 | M | - | - | + | - | - | - | - |
| E3 | M | - | - | - | - | - | - | - |
| E32 | F | - | - | - | + | + | - | - |
| E17 | M | - | - | + | - | - | - | + |
| E38 | F | - | - | + | - | - | - | - |
| E62 | F | - | - | - | - | - | - | - |
| E35 | M | + | - | - | - | - | - | - |
| E45 | F | - | + | + | - | - | + | - |
| E46 | M | - | - | - | - | - | - | - |
| E69 | F | + | - | - | - | - | - | - |
| E36 | M | - | - | - | - | - | - | - |
| E53 | F | + | - | - | - | + | - | - |
| E42 | M | - | - | - | - | - | - | + |
| E10 | M | + | - | - | - | - | - | - |
| E68 | M | + | - | - | + | - | - | - |
| E49 | M | + | - | + | - | - | - | + |
| E51 | M | + | - | - | - | - | - | - |
| E61 | F | - | - | - | - | - | - | - |
| E40 | M | + | + | + | - | + | - | - |
| E65 | M | - | - | + | - | - | - | - |
| E43 | F | - | - | + | - | - | - | - |
| E44 | F | + | + | + | + | - | + | - |
| E67 | F | - | - | + | - | - | - | - |
| E72 | M | - | - | - | - | - | - | - |
| E57 | F | - | - | - | - | - | - | - |
| E6 | M | + | + | + | - | - | - | - |
| E16 | F | - | + | - | - | - | + | - |
| E64 | M | - | - | + | - | - | - | - |
| E52 | M | - | - | - | - | - | - | - |
| E71 | F | + | - | + | - | - | + | - |
| E70 | M | + | - | - | - | - | - | - |
| E48 | M | + | - | - | + | - | - | - |
| E59 | F | - | - | + | - | - | - | - |
| E28 | M | - | - | - | - | - | - | - |
| E8 | F | - | - | + | - | - | - | - |
| E54 | M | + | - | - | + | + | - | - |
| E18 | F | - | - | - | - | - | + | - |
| E4 | M | - | - | + | + | + | + | - |
| E29 | M | - | - | - | - | - | - | - |
| E37 | M | - | - | - | - | - | - | + |
| E39 | F | - | - | - | - | - | - | - |
| E21 | M | - | - | - | - | - | - | - |
| E47 | F | - | - | - | - | - | - | - |
| E22 | M | - | - | - | + | - | - | - |
| E7 | F | + | - | - | - | - | - | - |
| E25 | F | - | - | - | - | - | - | - |
| E60 | F | + | - | - | - | + | - | - |
| E13 | M | - | - | - | - | - | - | - |
| E27 | M | - | - | - | - | - | - | + |
| E30 | F | + | - | - | + | - | - | - |
| E58 | F | - | - | - | - | - | - | - |
| E33 | M | + | - | - | - | - | - | - |
| E56 | F | + | - | + | - | - | + | - |
| E14 | M | + | - | - | + | - | - | - |
| E5 | F | - | - | - | - | - | - | - |
| E15 | F | - | - | - | - | - | - | - |
| E23 | F | - | - | - | - | - | - | - |

F, Female; M, Male

**Table S2. Demographic data of non-carriers of ESBL-producing *E. coli***

| Code | Unit | Gender | Antibiotic usage within the past 6 months | Hospitalization within the past 6 months | Underlying disease | Antibiotic usage within the past 6 months in the family members | Hospitalization within the past 6 months in the family members | Surgery | Traveling abroad |
| --- | --- | --- | --- | --- | --- | --- | --- | --- | --- |
| E73 | E-ICU | M | + | + | - | - | - | - | - |
| E74 | OP | F | - | - | - | + | - | - | - |
| E75 | OP | F | + | - | - | - | - | - | - |
| E76 | OP | F | - | - | - | - | - | - | - |
| E77 | G-ICU | M | - | + | + | - | - | + | - |
| E78 | E-ICU | M | - | - | - | - | - | - | - |
| E79 | G-ICU | F | - | - | - | - | - | - | - |
| E80 | E-ICU | M | + | - | + | - | - | - | - |
| E81 | E-ICU | M | + | - | - | - | - | - | - |
| E82 | E-ICU | F | - | - | + | - | - | - | - |
| E83 | G-ICU | M | + | - | - | - | - | - | - |
| E84 | G-ICU | F | - | - | + | - | - | - | - |
| E85 | G-ICU | M | - | - | - | - | - | - | - |
| E86 | G-ICU | F | + | - | - | - | - | - | - |
| E87 | OP | F | + | - | - | - | - | + | - |
| E88 | OP | F | + | - | - | - | - | - | - |
| E89 | OP | F | - | - | - | - | - | - | - |
| E90 | OP | M | - | - | + | - | + | - | - |
| E91 | OP | F | - | - | - | - | - | + | + |
| E92 | OP | F | + | - | - | - | - | - | - |

F, Female; M, Male; ICU, intensive care unit; OP, Outpatient; G-ICU, general intensive care unit; E-ICU, emergency intensive care unit.
